# P-1492. Modeling the Public Health Impact of Routine Vaccination of Infants in the United States Against Invasive Meningococcal Disease Caused by Serogroup B

**DOI:** 10.1093/ofid/ofaf695.1676

**Published:** 2026-01-11

**Authors:** Elise Kuylen, Oscar Herrera-Restrepo, Lucian Gaianu, Justyna Zawieja, Yaneth Gil Rojas, Zeki Kocaata, Hiral Shah

**Affiliations:** GSK, Wavre, Brabant Wallon, Belgium; GSK, Wavre, Brabant Wallon, Belgium; GSK, Wavre, Brabant Wallon, Belgium; Putnam Associates, Krakow, Malopolskie, Poland; Putnam Associates, Krakow, Malopolskie, Poland; GSK, Wavre, Brabant Wallon, Belgium; GSK, Wavre, Brabant Wallon, Belgium

## Abstract

**Background:**

Invasive meningococcal disease (IMD) is uncommon but can lead to severe sequelae and death. In the United States (US), IMD incidence is highest among infants aged < 1 year, with most cases caused by serogroup B (MenB). However, no vaccine protecting against MenB is currently approved and recommended for children aged < 10 years in the US.

**Methods:**

We developed a population-based static model to explore the public health impact of introducing MenB vaccination into the US routine infant vaccination program at 2, 4, 6, and 12 months (4-dose series) or 2, 4, and 12 months (3-dose series) of age. Population size and background mortality were sourced from 2024 US Census data and 2021 life tables, respectively. Data from the 2015–2019 Enhanced Meningococcal Disease Surveillance (EMDS) reports were used to project MenB incidence and case fatality rates without vaccination. Vaccine effectiveness and average vaccine duration of protection (DoP) were extracted from prior real-world data on IMD protection offered by the multicomponent MenB vaccine (4CMenB). MenB vaccine coverage for both series was assumed based on mean 2015–2019 coverage for the diphtheria, tetanus, pertussis, and polio vaccine (DTaP-IPV).

**Results:**

Over a 15-year time horizon, the implementation of a MenB 4-dose vaccination series prevented 139 MenB IMD cases among infants aged < 1 year (47% case reduction) and 70 cases among children aged 1–10 years (31% case reduction). A 3-dose series prevented 135 MenB IMD cases among infants aged < 1 year (46% case reduction) and 70 cases among children aged 1–10 years (31% case reduction). Considering either vaccination schedule under evaluation, 11 deaths among children aged < 11 years would be prevented (4-dose series: 40% decrease; 3-dose series: 39% decrease).

**Conclusion:**

In the US, the highest IMD incidence is in infants. In our model, MenB vaccination led to substantial reductions in IMD cases and deaths among infants and, due to the duration of protection, also among children. Implementing MenB vaccination for all US infants may positively impact public health by preventing IMD cases and deaths, reducing both acute and long-term IMD burden and potentially improving the quality of life of children and caregivers.

Funding: GSK VEO-000946

**Disclosures:**

Elise Kuylen, PhD, GSK: Employee|GSK: Stocks/Bonds (Public Company) Oscar Herrera-Restrepo, PhD, GSK: Employee|GSK: Stocks/Bonds (Public Company) Lucian Gaianu, MSc, GSK: Employee Justyna Zawieja, MSc, GSK: Employee of Putnam Associates, which was paid by GSK to conduct this study Yaneth Gil Rojas, MSc, GSK: Employee of Putnam Associates, which was paid by GSK to conduct this study Zeki Kocaata, PhD, GSK: Employee|GSK: Stocks/Bonds (Public Company) Hiral Shah, PhD, GSK: Employee|GSK: Stocks/Bonds (Public Company)

